# Are we ever aware of concepts? A critical question for the Global Neuronal Workspace, Integrated Information, and Attended Intermediate-Level Representation theories of consciousness

**DOI:** 10.1093/nc/niv006

**Published:** 2015-10-02

**Authors:** David Kemmerer

**Affiliations:** Department of Speech, Language, and Hearing Sciences, Purdue University and Department of Psychological Sciences, Purdue University

**Keywords:** contents of consciousness, theories and models

## Abstract

To locate consciousness in the flow of synaptic activity in the brain, we must first locate it in the flow of information processing in the mind. Two different positions have been debated for centuries. The liberal view maintains that the contents of experience include not only sensory, motor, and affective states, but also concepts and the thoughts they enter into. In contrast, the conservative view maintains that concepts have no intrinsic qualia of their own, and that the contents of experience are therefore restricted to sensory, motor, and affective states. Here I discuss how this long-standing controversy is relevant to several contemporary neuroscientific theories of consciousness. I do so, however, in a manner that is admittedly biased toward the conservative view, since I am among those who believe that it is more consistent than the liberal view with a number of key findings. I focus first on two of the most prominent neuroscientific theories of consciousness—namely, Stanislas Dehaene's Global Neuronal Workspace Theory and Giulio Tononi's Integrated Information Theory. I argue that because both of these approaches assume the liberal view, they are challenged in significant ways by data favoring the competing conservative view. I then turn to a third framework—namely, Jesse Prinz's Attended Intermediate-Level Representation Theory. I contend that because it explicitly endorses the conservative view, it has a unique advantage over the other two approaches. I also point out, however, that it has independent shortcomings that prevent it from achieving adequate explanatory coherence. I conclude by emphasizing that, if the conservative view is in fact correct, a central goal of future research should be to distinguish, at both psychological and neurobiological levels of analysis, between the following two kinds of information processing that often occur simultaneously: first, activation of the modality-specific sensory, motor, and affective representations that constitute the sole ingredients of conscious experiences; and second, activation of the conceptual representations that give those experiences meaning and that may even influence them in a top-down manner, but that never themselves reach awareness.

## Introduction

Research on consciousness has made numerous advances during the past 25 years (Boly *et al*., 2013; Block *et al*., 2014). However, apart from a few notable exceptions (Miller, 2007; Melloni and Singer, 2010; Aru *et al*., 2012b; de Graaf *et al*., 2012; Prinz, 2012; Pitts *et al*., 2014a), progress in elucidating the *neural* correlates of consciousness has been hampered by a lack of sufficient attention to the *cognitive* correlates of consciousness—that is, to the forms of mental representation that actually reach awareness. This is a critical issue, since the boundaries of what have been called “the admissible contents of experience” (Hawley and Macpherson, 2011) necessarily impose strict constraints on theories of how and why the brain generates consciousness.

Although this issue has not been adequately addressed in the neuroscientific literature, it has been the focus of intense debate in the philosophy of mind and related fields (for a recent collection of papers, see Bayne and Montague, 2012a). The main controversy revolves around the question of whether it is possible for the highest levels of mental representation—in particular, concepts and the thoughts they enter into—to ever achieve consciousness when activated. The two most frequently distinguished positions are the liberal and conservative views, both of which are summarized below. (A terminological note: Throughout this article, the words *consciousness, awareness,* and *experience* are used interchangeably.)

### The liberal view

The central claim of the liberal view is that although concepts do not always reach awareness when activated, they frequently do, and in those situations they bring about a particular kind of experience that cannot be reduced to any form of verbal or nonverbal imagery but is instead distinctively cognitive in nature. This unique type of awareness is sometimes called “cognitive phenomenology” or “cognitive qualia,” and it ostensibly cuts across the entire spectrum of mental activities that employ concepts, including thinking and reasoning, producing and comprehending language, and categorizing objects and events in the world. As Bayne and Montague (2012b) point out, the liberal view has a long and illustrious history, having been endorsed by such luminaries as René Descartes, John Locke, George Berkeley, David Hume, Franz Brentano, Edmund Husserl, Immanuel Kant, William James, and G.E. Moore. In the early 20th century, the core members of the famous Würzburg school of psychology—namely, Oswald Külpe, Narziss Ach, and Karl Bühler—claimed to have discovered introspective evidence for a pure form of cognitive phenomenology that they called non-imagistic thought. And more recently, a number of philosophers have upheld the position that consciousness is not limited to the senses but encompasses high-level semantic knowledge too. Here are a few representative examples, mostly drawn from Bayne and Montague (2012b):

In addition to arguing that there is something it is like to think a conscious thought, I shall also argue that what it is like to think a conscious thought is distinct from what it is like to be in any other kind of conscious mental state, and that what it is like to think the conscious thought that *p* is distinct from what it is like to think any other conscious thought … (Pitt, 2004, p. 2)

Intentional states have a phenomenal character, and this phenomenal character is precisely the what-it-is-like of experiencing a specific propositional-attitude vis-à-vis a specific intentional content. Change either the attitude-type (believing, desiring, wondering, hoping, etc.) or the particular intentional content, and the phenomenal character thereby changes too. (Horgan and Tienson, 2002, p. 522)

… generally, as we think—whether we are speaking in complete sentences, or fragments, or speaking barely or not at all, silently or aloud—the phenomenal character of our noniconic thought is in continual modulation, which cannot be identified simply with changes in the phenomenal character of either vision or visualization, hearing or auralization, etc. (Siewert, 1998, p. 282, emphasis suppressed)

When I am now phenomenally aware of the telephone on my desk, I am aware of it *as a telephone* and as located at a particular place in my world … .Introspectively, all of this information is experienced as at least implicitly present as part of the phenomenal content of my perceptual state. (van Gulick, 1994, p. 34, emphasis added)

The liberal view is elaborated and defended more fully in the following additional references: Strawson (1994, 2012), Siegel (2005), Bayne (2009), Horgan (2012), Nes (2012), Pitt (2012), Shields (2012), Siewert (2012), Woodruff-Smith (2012), Jorba and Vicente (2014), and Chudnoff (2015).

### The conservative view

In sharp contrast to the liberal view, the conservative view maintains that we are never directly aware of concepts *per se;* instead, consciousness is always restricted to sensory, motor, and affective states. Proponents of this position typically argue that even though it sometimes seems as if our thoughts are conscious, this is just an illusion that stems from the tendency to mistakenly treat the verbal and nonverbal images that often accompany certain thoughts as being equivalent to the conceptual contents of those thoughts. Advocates of the conservative view also believe that while concepts can certainly influence perception (Hansen *et al*., 2006; Meteyard *et al*., 2007; Gendron *et al*., 2012; Lupyan and Ward, 2013), we only experience the sensory effects of such top-down processes, never the high-level causes. Bayne and Montague (2012b) note that just as the liberal view has a rich historical background, so does the conservative view. Challenging the Würzburg psychologists mentioned above, both Wilhelm Wundt and Edward Titchener contended that introspection provides no convincing evidence that thought can be manifested in consciousness by itself—that is, independent of modality-specific imagery. Similarly, the notion that concepts have intrinsic qualia has been vigorously attacked by such famous philosophers as J.J. Smart, Gilbert Ryle, and Hilary Putnam. Moving closer to the present, during the past few decades many theorists have continued to challenge the notion of cognitive phenomenology, as illustrated by the following passages, mostly drawn from Bayne and Montague (2012b):

Should we include any mental states that are not feelings and experiences on the list of phenomenally conscious states? Consider my desire to eat ice cream. Is there not something it is like for me to have this desire? If so, is this state not phenomenally conscious? And what about the belief that I am a very fine fellow? Or the memory that September 2 is the date on which I first fell in love? … . It seems to me not implausible to deal with these cases by arguing that insofar as there is any phenomenal or immediately experienced felt quality to the above states, this is due to their being accompanied by sensations or images or feelings that are the real bearers of the phenomenal character. (Tye, 1995, p. 4)

As best we can tell, believing that 17 is a prime number does not feel any different from believing that 19 is a prime number. Indeed, as best we can tell, neither of these states has any distinctive qualitative properties. Neither of them feels like much at all. (Nichols and Stich, 2003, p. 196)

Bodily sensations and perceptual experiences are prime examples of states for which there is something it is like to be in them. They have a phenomenal feel, a phenomenology, or, in a term sometimes used in psychology, raw feels. Cognitive states are prime examples of states for which there is *not* something it is like to be in them, of states that lack a phenomenology. (Braddon-Mitchell and Jackson, 2007, p. 129, original emphasis)

I will argue … that the felt qualities of our thoughts can be completely accommodated by appeal to concomitant sensory imagery. (Prinz, 2012, p. 149)

An iconoclastic way of putting this would be to say that there really are no such things as conscious thoughts … .(Jackendoff, 2012, p. 84)

The conservative view is elaborated and defended more fully in the following additional references: Levine (1983), Jackendoff (1987),Lormand (1996), Clark (2000), Langsam (2000), Wilson (2003), Carruthers (2005), Robinson (2005, 2012), Damasio (2010), O'Callaghan (2011), Carruthers and Veillet (2012), and Tye and Wright (2012).

### Aims

The overarching goal of this article is to show that the controversy over whether we are ever aware of concepts is quite relevant to research on the neural substrates of consciousness. Here at the outset, I must acknowledge that space limitations prevent me from presenting a thorough, well-balanced summary of the debate, let alone attempting to resolve it. I do, however, believe that the conservative view has more empirical support than the liberal view, and for this reason I will concentrate on describing some ways in which the former approach has serious implications for several contemporary neuroscientific theories of consciousness. Two of the most prominent theories—namely, the Global Neuronal Workspace Theory (GNWT) (Dehaene and Naccache, 2001; Dehaene and Changeux, 2011; Dehaene, 2014; Dehaene *et al*., 2014) and the Integrated Information Theory (Tononi, 2004, 2008, 2012a, 2012b; Oizumi *et al*., 2014; Tononi and Koch, 2015)—clearly adopt the liberal view, but they do so without overt justification and without discussing the alternative position. Therefore, in the first two sections I take up each theory in turn, highlight a number of the chief proponents' assumptions and assertions that reflect the liberal view, and raise counter-arguments from the perspective of the conservative view. Then in the next section I summarize another theory—namely, the Attended Intermediate-Level Representation Theory (Prinz, 2012)—and contend that it warrants greater consideration because it not only endorses the conservative view but has several other strengths as well. I also point out, however, that it has independent shortcomings that prevent it from achieving adequate explanatory coherence. Finally, I conclude by emphasizing the need for researchers in the neuroscience of consciousness to realize that the question of whether concepts ever reach awareness is a significant one with major consequences for both theoretical frameworks and experimental investigations.

## The Global Neuronal Workspace Theory (GNWT)

### Summary of the GNWT

Building on a previous proposal by Baars (1988), the GNWT developed by Dehaene and colleagues holds that consciousness arises from a capacity-limited architecture that is adaptively designed to extract relevant information from a variety of mental systems and make it broadly available for purposes such as linguistic encoding, memory storage, planning, and decision-making (Dehaene and Naccache, 2001; Dehaene and Changeux, 2011; Dehaene, 2014; Dehaene *et al*., 2014). During perception, a massive amount of information is processed unconsciously by specialized mechanisms that operate in parallel. Some of that information, however, is selected as being especially pertinent to the individual's present goals, and it then crosses the threshold of conscious access and enters the global workspace for flexible sharing. According to Dehaene (2014, p. 168), “this global availability of information is precisely what we subjectively experience as a conscious state.”

Based on many experiments involving minimal contrasts between conscious and unconscious conditions, the GNWT maintains that conscious access has four neuronal signatures. First, starting roughly 300 ms after stimulus onset, there is a sudden ignition of activity that includes not only the regions that represent the specific content (e.g., color, shape, motion, etc.) of the given conscious state, but also the regions that comprise the backbone of the global workspace—namely, the dorsolateral prefrontal cortex, the anterior cingulate cortex, the inferior parietal cortex, and the precuneus, all of which form a so-called “rich club” network of tightly interconnected hubs (van den Heuvel and Sporns, 2011; van den Heuvel *et al*., 2012). Second, this widely distributed ignition is accompanied by a P300 event-related potential (ERP) component. Third, in conjunction with the P300 wave, there is a significant increase in high-frequency gamma-band (> 30 Hz) oscillations in the ignited regions. Finally, during the same time window, and with the help of thalamocortical loops, these regions become functionally integrated as a transiently stable coalition or “brain web” through synchronized reciprocal signals carried by long-distance excitatory axons.

### Critique of the GNWT

Although the GNWT has many virtues, its putative signatures of consciousness are inconsistent with several empirical findings. For instance, although the dorsolateral prefrontal cortex is necessary for monitoring and maintaining perceptual information, it does not appear to be essential for consciously experiencing that information (Penfield and Evans, 1935; Mataró *et al*., 2001; Rounis *et al*., 2010; Frässle *et al*., 2014). In addition, there is evidence that the P300 wave and enhanced gamma oscillations reflect separate task-related post-perceptual processes, rather than the process of becoming aware of stimuli (Pitts *et al*., 2014a, 2014b).

Here, however, I focus on a different aspect of the GNWT—specifically, that it assumes, in line with the liberal view, that consciousness is not restricted to sensory, motor, and affective representations, but encompasses concepts as well. To be sure, Dehaene not only acknowledges, but has played a major role in demonstrating, that concepts such as those denoted by numerals, words, and phrases can be processed outside of awareness (Dehaene *et al*., 1998, 2001; Naccache and Dehaene, 2001; Kouider and Dehaene, 2007; Van Gaal *et al*., 2014). But it is also quite clear from his recent book, *Consciousness and the Brain* (Dehaene, 2014), that he believes that concepts, and the thoughts they enter into, are perfectly viable candidates for participating in the global workspace of experience, and that they actually serve important cognitive functions within that workspace. Indeed, the subtitle of his book is *Deciphering How the Brain Codes Our Thoughts,* and the expression “conscious thought” occurs at least half a dozen times throughout its pages (pp. 20, 53, 110, 146, 175, 251), including in the title of Chapter 4, “The signatures of a conscious thought.” In what follows, I consider five parts of the book where this notion of conscious thought is discussed, and in each case I argue that it is inconsistent with evidence which supports the opposite conservative view that thoughts are always hidden from awareness.

To begin, Dehaene (2014, pp. 145–48) summarizes several intracranial recording studies that have revealed single neurons in the human anterior temporal lobes (ATLs) that respond fairly selectively to pictures of particular entities, including famous people like Bill Clinton and Jennifer Aniston, as well as famous locations like the Sydney Opera House and the World Trade Center (Kreiman *et al*., 2000, 2002; Quiroga *et al*., 2005, 2008a, 2008b). When a patient sees different pictures of the same person or place—for example, assorted photographs, portraits, and line drawings of Bill Clinton—the firing rate of the relevant cell reliably tracks the invariant identity of that object, regardless of radical shifts in the fine-grained features of the images. Moreover, the cell's firing rate also follows the patient's reports of the visibility of the stimuli. In commenting on these remarkable findings, Dehaene states that what the discharge patterns index “is neither a global arousal signal nor myriad changing details, but the gist of the current picture—just the right sort of stable representation that we would expect to encode our conscious thoughts.” In the same vein, he also states that when we observe such patterns, “we are witnessing the contents of consciousness.”

These ATL cells do appear to contribute substantially to our conceptual knowledge of well-known people and places; indeed, that is why Quiroga (2012) calls them “concept cells.” But the mere fact that their activation correlates with conscious experiences of these entities does not imply that their representational contents are an inherent part of such experiences (Aru *et al*., 2012b). On the contrary, two important points suggest that, in keeping with the conservative view, the concepts captured by these cells never reach awareness.

The first point involves the contrast between the remarkable specificity of experiences and the equally remarkable generality of concepts. Perhaps the most salient property of conscious states is their extraordinary degree of differentiation. Even if we restrict our attention to the visual domain, it is obvious that the number of potentially separate experiences is exceedingly large, limited only by one's imagination. The primary function of concepts, however, is to abstract away from all of this diversity so that certain aspects of experiences can be regarded as instances of more wide-ranging, similarity-based categories. In fact, according to the conservative view, it is precisely because concepts always transcend the experiences they apply to that they always remain unconscious. Both Prinz (2012) and Jackendoff (2012) underscore this point:

When I look at a chair, try as I may, I only see a specific chair oriented in a particular way. … it's not clear what it would mean to say that one visually experiences chairness. What kind of experience would that be? A chair seen from no vantage point? A chair from multiple vantage points overlapping? A shape possessed by all chairs? Phenomenologically, these options seem extremely implausible. (Prinz, 2012, p. 74)

Now the interesting thing is that everything you *perceive* is a particular individual (a token)—you can't perceive categories (types). And you can only *imagine* particular individuals—you can't *imagine* categories. If you try to imagine a type, say forks in general, your image is still a particular fork, a particular token. (Jackendoff, 2012, p. 130)

In the current context, what matters most is that this line of argumentation is not restricted to concepts for types of entities, like chairs and forks, but also applies to concepts for one-of-a-kind entities, like the well-known people and places that are represented by the sorts of ATL cells described above. Even though unique entities like these are significant enough to warrant dedicated concepts, they may still be consciously experienced in a vast if not infinite number of ways. For instance, non-living things such as eminent landmarks can be seen under different lighting conditions, at different distances, from different angles, etc., and living things such as famous people can vary in their appearance even more, due to changes associated with facial expression, posture, hairstyle, age, etc. But, while the specific ways that certain unique entities *look* to us may radically shift across different situations, the concepts that enable us to identify them remain relatively invariant. Moreover, and most critically, the activation of these concepts during the recognition process does not seem to add anything distinctively cognitive to the experience. For example, to reformulate Prinz's (2012, p. 74) statement, it is not clear what it would mean to experience “Bill Clinton-ness” or “Jennifer Aniston-ness” in some sort of purely conceptual sense that goes beyond perceptual images. These observations support the hypothesis that, contrary to Dehaene's (2014, pp. 145–48) proposal, the concepts encoded by ATL cells never achieve consciousness; instead, they always perform their work beneath the surface of awareness.

The second point involves a set of neuropsychological studies that provide further evidence for this position. In particular, it has been repeatedly shown that dysfunction of the ATLs due to stroke, surgical resection, gradual deterioration, or congenital disease impairs the ability to recognize famous people like Bill Clinton and Jennifer Aniston, but does not impair the ability to consciously see their faces (A.R. Damasio *et al*., 1990; H. Damasio *et al*., 2004; Snowden *et al*., 2004; Avidan *et al*., 2014). This dissociation greatly strengthens the argument that whenever we see a familiar person, the relevant concept cells in the ATLs play an essential role in allowing us to identify them, but do not contribute directly to our conscious experience. Instead, the experience itself appears to be subserved by large assemblies of cells in the intermediate regions of the ventral face processing network—that is, the occipital face area and the fusiform face area—that represent the detailed visual features of faces (Tsao *et al*., 2008; Axelrod and Yovel, 2013; Von Der Heide *et al*., 2013; Rangarajan *et al*., 2014).

Now, a critic, especially one who espouses the liberal view, might say that because perception is not driven entirely by bottom-up input, but is instead modulated by predictions and other kinds of prior representational states (Kosslyn, 1994; Friston, 2005; Clark, 2013; Panichello *et al*., 2013), it is certainly possible that in some situations one's concept of how a familiar person typically looks does in fact influence one's conscious experience of seeing them, if only in subtle ways. If that were the case, however, it would not imply that one is actually aware of the concept itself; rather, it would only imply that one is aware of the top-down effects of that concept (for relevant findings, see Hansen *et al*., 2006; Meteyard *et al*., 2007; Gendron *et al*., 2012; Lupyan and Ward, 2013).

Returning to Dehaene's (2014, pp. 99–100) book, he elaborates the GNWT by comparing consciousness to the spokesperson of a large institution who voices the “common wisdom” extracted from different departments of a complex staff composed of thousands of specialist employees. While developing this analogy, he states that “like a presidential brief, the brain's conscious summary must contain an interpretation of the environment written in a ‘language of thought' that is abstract enough to interface with the mechanisms of intention and decision making.” How, though, could any level of representation that is abstract enough to bridge the gap between perception and behavior have any intrinsic qualia of its own, over and above the types of modality-specific qualia that are associated with the sensory and motor representations that must be connected with each other? Dehaene does not attempt to answer this question, nor does he acknowledge that it is a genuine issue. He simply adopts the liberal view that the concepts comprising the “language of thought” can reach awareness when activated, without either justifying that perspective or discussing the opposite conservative view that concepts always operate unconsciously.

Later in the same chapter, Dehaene (2014, p. 110) expands on the notion that consciousness is like a summary of relevant information by stating that it includes “a multisensory, viewer-invariant, and durable synthesis of the environment.” But neither visual awareness nor any other form of experience contains viewer-invariant representations; on the contrary, possessing a first-person perspective—one that, for sighted people, is typically anchored behind the eyes—is often taken to be a fundamental requirement of bodily self-consciousness (Blanke and Metzinger, 2009). This is quite pertinent to the main topic of this article because, according to the conservative view, one of the reasons why concepts cannot reach awareness is because they always generalize over particular perspectives. This key insight is nicely captured by Prinz (2012, p. 74) in the passage quoted earlier, where he makes what is essentially the following argument: the concept of a chair is viewer-invariant, which is to say that it covers all possible vantage points; however, it is impossible to see or imagine a chair “from no vantage point” or “from multiple vantage points overlapping”; therefore, it is impossible to directly experience the concept of a chair, that is, “chairness” in the most general sense.

In another part of his book, Dehaene (2014, pp. 177–78) uses the example of Leonardo da Vinci's *Mona Lisa* to illustrate his idea that a conscious state is underpinned by millions of widely distributed neurons that represent different facets of the experience and that are functionally integrated through bidirectional, rapidly reverberating signals. Most importantly for present purposes, he claims that when we look at the classic painting, our global workspace of awareness includes not just its visual properties (e.g., the hands, eyes, and “Cheshire cat smile”), but also “fragments of meaning,” “a connection to our memories of Leonardo's genius,” and “a single coherent interpretation,” which he characterizes as “a seductive Italian woman.” This part of the book clearly reveals Dehaene's endorsement of the liberal view that concepts are among the kinds of information that can reach consciousness. The problem, however, is that he does not explicitly defend this position against the opposite conservative view, which denies that we can directly experience complex semantic structures like the one expressed by the phrase “a seductive Italian woman.” The meaning of the word *seductive,* for instance, is highly abstract, since it applies not only to the nature of Mona Lisa's smile, but also to countless other visual and non-visual stimuli that satisfy the conceptual criteria of, to quote from Webster's dictionary, “having tempting qualities.” On the one hand, it is reasonable to suppose that there is something it is inimitably like, phenomenologically speaking, to perceive particular instances of seductive stimuli, such as Mona Lisa's smile. But on the other hand, it is extremely hard to imagine how anyone could directly experience seductiveness in some sort of general, all-encompassing sense. Hence, the conservative view maintains that this concept, like all others, lacks intrinsic qualia.

Near the end of his book, Dehaene (2014, p. 251) proposes that “the human global neuronal workspace may be unique in its capacity to formulate conscious thoughts such as ‘taller than Tom,' ‘left of the red door,' or ‘not given to John.'” Once again, though, advocates of the conservative view could argue that while the phonological forms of these phrases can certainly reach awareness, their compositional meanings are far too abstract to do so. For example, the expression *taller than Tom* encodes a comparative scalar relationship that could apply to an infinite number of entities whose extent along the dimension of height exceeds that of Tom but is otherwise unbounded (Bierwisch and Lang, 1989). One could generate a vast array of conscious mental images that depict this relationship in various ways, but none of them would be able to indicate, in a purely visual, non-symbolic fashion, that what's really important is the relative height of the two objects. Turning to the expression *left of the red door,* it refers to a region of space that is determined in two steps: first, the left and right sides of the door are identified as the ones that correspond, in a mirror-reflecting manner, to the left and right sides of the viewer facing the door; and second, the target domain is specified by projecting out a moderate distance in the leftward direction defined by the horizontal left/right axis imposed on the door (Levinson, 2003). As with the expression *taller than Tom,* it would be impossible for any particular image to indicate in a non-symbolic fashion exactly how this spatial relationship is determined. It also bears mentioning that any conscious representation of the details of the situation described by the phrase *left of the red door* would need to portray the door as having a precise shape and a precise shade of red, thereby making the image in awareness much more specific than the actual content of the linguistically encoded concepts. Finally, the expression *not given to John* refers to an unrealized event, and for this reason it is quite difficult, to say the least, to understand how such a semantic structure could ever be directly experienced. What could it possibly be like to consciously represent the concept of negation in a way that fully complies with its extraordinary degree of abstractness (Horn, 1989)?

Before concluding this discussion of the GNWT, it is worthwhile to briefly invoke Block's (1995) distinction between phenomenal consciousness and access consciousness and ask whether Dehaene (2014) might regard concepts, and the thoughts they enter into, as being available to awareness in the latter sense rather than the former. In the last chapter of his book, Dehaene (2014, p. 261) does mention some of Block's work, but unfortunately he does not address the distinction that Block proposed, so it is difficult to know where he stands on this issue. Nevertheless, some evidence that he might regard activated concepts as being potentially conscious in the access sense rather than the phenomenal sense comes from the fact that he frequently uses the expression “conscious access” not only in his book but also in other presentations of his theory (Dehaene and Naccache, 2001; Dehaene and Changeux, 2011; Dehaene *et al*., 2014).

It is important to note, however, that Block's (1995) distinction is by no means uncontroversial, and some proponents of the conservative view reject it. Prinz (2012, p. 6) is one such scholar, as revealed by his remark that “information access seems conscious … when and only when it is accompanied by phenomenal experience.” With respect to concepts, their retrieval is often accompanied by inner speech, visual images, and other forms of phenomenal experience, but the activated concepts themselves do not appear to have any uniquely cognitive qualia of their own, and this suggests that access consciousness may not exist as a special kind of awareness that is separate from phenomenal consciousness.

Consider, for example, the tip-of-the-tongue state, which occurs when you have accessed a particular concept—say, the one encoded by the word *pterodactyl*—but cannot recall its name. While searching your memory for this elusive word, you might conjure up various kinds of verbal and nonverbal imagery, like saying to yourself “It's a large flying dinosaur” and visualizing the appearance of the fearsome creature. But, as indicated above, these forms of modality-specific imagery do not actually constitute the concept, since the concept itself resides at a higher level of generalization. The key question, then, is this: apart from such imagery, and from the frustrating sense of persistently groping for the desired word, what is left in your conscious awareness? According to the conservative view, nothing. Even though the meaning of the word continues to be activated, it does not have any inherent qualia of its own.

A similar situation involves the realization that what one has just said does not accurately express what one was trying to say. Besides showing that thought is independent of language, such events provide additional evidence for the conservative view, as Jackendoff (2012, pp. 90–91) explains:

… we can only be aware of the content of our thoughts if they're linked with pronunciation. So if we haven't yet turned a thought into words, we're only aware at best of *thinking going on,* not of exactly what the thought *is.* If we then utter a sentence, we can unconsciously compare the thought it expresses with the thought we intended to express, and we can get the feeling that the utterance is inadequate.

To summarize, the GNWT adopts the liberal view that activated concepts not only can but often do reach awareness. Hence it is at odds with numerous empirical findings and theoretical arguments that favor the competing conservative view that concepts never reach awareness. This is arguably a significant limitation of the theory. Perhaps the most serious consequence is that the theory's characterization of the neuronal signatures of consciousness may turn out to be too broad. This is because over the course of their research on these signatures, Dehaene and his colleagues have not taken care to distinguish between, on the one hand, the sorts of sensory, motor, and affective representations that can occur in conscious experiences, and on the other hand, the sorts of high-level conceptual representations that—again, according to the conservative view—cannot.

Suppose, for example, that one suddenly saw a bicycle. According to the GNWT, the shapes, sizes, colors, and spatial arrangements of the object's parts would enter one's awareness after roughly 300 ms, and the resulting conscious state would be subserved by the synchronization of enhanced gamma oscillations in many populations of neurons distributed across not only certain visual areas of the brain, but also other areas involved in processes such as attention, short-term memory, and the widespread broadcasting of information. Now, assuming that one is familiar with bicycles, one's concept of a bicycle would also be activated—specifically, in a well-studied tool-related network consisting of certain temporal, parietal, and frontal areas (Garcea and Mahon, 2014; Stevens *et al*., 2015)—and the activation of that concept would allow one to rapidly recognize the object and draw inferences about it (Grill-Spector and Kanwisher, 2005). This process of concept retrieval, however, would, according to the conservative view, take place unconsciously. So we are left with the following question: How exactly would the neural correlates of the *conscious perception* of the bicycle differ from the neural correlates of the *unconscious recognition* of the bicycle? This issue has yet to be rigorously investigated, but it must ultimately be resolved if genuine progress is to be made in the neuroscience of consciousness—once more, assuming the conservative view is actually correct.

## The Integrated Information Theory (IIT)

### Summary of the IIT

Originating from a previous proposal called the Dynamic Core Hypothesis (Tononi and Edelman, 1988; Edelman and Tononi, 2000), the IIT has been developed primarily by Tononi as a mathematical approach to measuring both the quantity and the quality of consciousness not only in biological organisms such as ourselves, but also, at least in principle, in artificial devices such as robots (Tononi, 2004, 2008, 2012a, 2012b; Oizumi *et al*., 2014; Tononi and Koch, 2015). The IIT highlights the fact that a conscious state is simultaneously differentiated (i.e., every experience is unique insofar as it rules out a tremendous number of alternative possibilities) and integrated (i.e., every experience comprises a unified “scene” perceived from a particular perspective). It therefore predicts that variable degrees of awareness will be associated with variable degrees of differentiation and integration in the human thalamocortical system. Although this idea requires further refinement, it has been supported by studies involving not only healthy adults at different stages of the sleep–wake cycle (Massimini *et al*., 2005), but also brain-damaged patients in either vegetative or minimally conscious states (Casali *et al*., 2013). These studies employed complex analyses of neural activity that were indirectly based on the central construct of the IIT, namely a formula referred to as “phi” (Φ), which is postulated to be a marker of consciousness, since it measures the amount of differentiated and integrated information in a system composed of multiple parts.

Concepts are major ingredients of the IIT, but they are defined in a technical manner as discrete mechanisms that are anatomically implemented by (sets of) neurons and that functionally specify irreducible cause–effect repertoires, where in each case the *cause* is the set of past inputs that give rise to the present on/off state of the mechanism, and the *effect* is the set of future consequences that follow from the present on/off state of the mechanism. Tononi maintains that such mechanisms are organized as nested hierarchies throughout the cerebral cortex, from the very lowest levels of representation to the very highest. In his scientific papers, most of the examples of this part of the framework are rather dense, but in his recent book, *Phi: A Voyage from the Brain to the Soul* (Tononi, 2012b), he presents a literary version of his theory, and in Chapter 19, he includes a fairly clear discussion of what he means by concepts. For instance, he writes that “we may discover a mechanism for detecting light in the center, another one for light on the left side, one for blue and one for red, one for oval and one for square shapes; one for noses, one for lips, and one for faces, and maybe even one for her, whoever she might be” (Tononi, 2012b, p. 201). All of these mechanisms are assumed to be separate concepts implemented by (sets of) neurons at different levels of the visual hierarchy, from V1 all the way up to the ATLs. Interestingly, Tononi argues that no single neuron, and hence no single concept, has any meaning independent of the various networks of mechanisms in which it is embedded, because the representational content of each one can only be identified by its relationship with, and especially its differentiation from, the others. He also argues, however, that whenever a multitude of concepts are coactivated in such a way that they collectively yield an irreducible cause–effect repertoire, the entire assembly constitutes a multifaceted “conceptual structure.” And this in turn leads to what is, for present purposes, the most important aspect of the theory—specifically, its strong endorsement of the liberal view that concepts are conscious when activated.

This key assumption is explicitly formalized in the IIT as the “central identity” thesis, which asserts that “an experience is a maximally integrated conceptual (information) structure or quale—that is, a maximally irreducible constellation of points in qualia space” (Tononi, 2012a, p. 306). Indeed, according to the framework, at any given time whatever conceptual structure happens to be activated in an intricate information processing system such as a human brain “completely specifies ‘what it is like to be' that particular mechanism in that particular state” (Tononi, 2012a, p. 306). Tononi and Koch (2015) elaborate this crucial claim by imagining a situation in which one watches a movie starring Jennifer Aniston (JA), and it is clear from their discussion that they believe one's experience would consist of a rapidly shifting series of fantastically complicated conceptual structures implemented by enormous neural networks distributed across the thalamocortical system. Some of the elements of these putative conceptual structures would change their on/off status quite frequently, like those subserved by the low-level cells in V1 that specify the orientations of edges in certain parts of the visual field. Other elements, however, would remain engaged for several seconds or even several minutes, like “the invariant concept ‘JA's face',” which is presumably subserved by high-level cells in the ATLs (Tononi and Koch, 2015, p. 9). In short, the IIT equates consciousness with concepts, regardless of their degree of complexity.

### Critique of the IIT

Although this approach has many merits, it also has some serious weaknesses. In the current context, the most salient problem is that the IIT is incompatible with data supporting the conservative view that we are never aware of concepts. For instance, as argued above in connection with Dehaene's GNWT, even though one's concept of a famous person like Jennifer Aniston is activated whenever one sees, hears, or thinks about her, this does not entail that the concept itself is part of those experiences (Aru *et al*., 2012b). On the contrary, one's conscious perception of this particular actress in TV shows, movies, magazines, etc., would probably not be significantly different (apart from changes in the associated verbal and nonverbal imagery, and perhaps the lack of subtle top-down effects) if one did not even recognize her due to having never learned her name and background or to having lost that knowledge as the result of ATL damage (A.R. Damasio *et al*., 1990; H. Damasio *et al*., 2004; Snowden *et al*., 2004; Avidan *et al*., 2014). This is because at any given moment one's awareness of Jennifer Aniston (or of anyone else, for that matter) is shaped not so much by one's degree of familiarity with that unique individual, but rather by such idiosyncratic and transient factors as whether one sees them from the front, the left, the right, half hidden behind a chair or table, sitting, standing, yawning, stretching, in candlelight, under a street lamp, through a fog, and so on (Millikan, 2014). According to the conservative view, the contents of consciousness consist of modality-specific details like these, not the high-level concepts that generalize over them.

A similar line of criticism also applies to Tononi's treatment of concepts of non-unique entities. For example, in Chapter 19 of *Phi,* one of the characters talks about how the machinery of conceptual representation could create “the idea of a triangle, wherever it may be, no matter how large or small, no matter where its corners are pointing, no matter whether equilateral, isosceles, or scalene” (Tononi, 2012b, p. 200). Even a concept as abstract as this would, according to the IIT, be conscious when activated. But how could the notion of a triangle possibly have any distinctive qualia? How could it ever be directly experienced? After all, no image could capture the conceptually vital fact that, as Tononi's character observes, a triangle must have three sides but need not have any particular size or shape. Similarly, Jackendoff (2012, p. 52) points out that nothing in a particular image of a triangle tells us that “having three sides is what's important for trianglehood.” And he goes on to note that “once you state that as the critical feature, you've gone outside of what visual images can do.”

In closing, the main message is that Tononi's IIT has the same significant limitation as Dehaene's GNWT. It assumes—incorrectly, according to the conservative view—that concepts can reach awareness when activated, and as a consequence its account of the neural underpinnings of consciousness appears to be too inclusive.

## The Attended Intermediate-Level Representation Theory (AIRT)

Expanding on earlier work by Jackendoff (1987), the AIRT developed by Prinz (2012) stands in sharp contrast to both the GNWT and the IIT because it adopts at the very outset the conservative view that we are never aware of concepts. In fact, based on the strength of the evidence for this view, Prinz (2012, p. 32) maintains that “an adequate theory should restrict consciousness to processes that lie outside of those systems that underwrite our highest cognitive capacities.” Because the AIRT is among the few contemporary frameworks, if not the only one, that achieves this goal, its three major tenets are worth summarizing and evaluating here.

First, a central claim is that perceptual awareness arises at intermediate rather than low or high levels of sensory hierarchies. In the visual domain, for example, what we experience is a world of vividly colored objects with clear contours, located at different distances from us and framed by our own point of view. Such conscious states do not correspond to the “flat, disunified jumble” (Prinz, 2012, p. 51) that is encoded in V1, nor do they correspond to the abstract, viewer-invariant concepts that are encoded in high-level regions of the temporal, parietal, and frontal lobes. Rather, they correspond to the kinds of attribute-specific representations that are constructed by specialized cortical areas at more intermediate stages of the visual system. Exactly which areas are part of this privileged family is not yet clear, but Prinz suggests that likely candidates include V2, V3, V3A, V4, V5, V6, and V7, since they have been linked with the awareness of form, color, motion, depth, and perspective.

Second, the AIRT maintains that intermediate-level representations only become conscious when they are modulated by attention and thereby made available to working memory. Although the precise relationship between consciousness and attention has been, and continues to be, quite controversial, Prinz marshalls a substantial amount of psychological and neurobiological evidence to support his hypothesis. He also emphasizes that while conscious information must always be *accessible* to working memory, it need not always be *accessed* to working memory—as, for instance, when one only glimpses a tiny flash of light for a few milliseconds and does not subsequently reflect on that experience. This is an important point because it distinguishes the AIRT from the GNWT. As indicated above, the GNWT assumes that in order for information to reach awareness, it must be brought into the global workspace—that is, into the large-scale storage and broadcasting system that includes resources for working memory in the lateral prefrontal cortex. The AIRT rejects this requirement, however, and it is therefore more compatible than the GNWT with data suggesting that consciousness can occur without prefrontal involvement (e.g., see Kouider *et al*., 2007, and the comments on that study by Prinz, 2012, p. 31; see also Penfield and Evans, 1935; Rounis *et al*., 2010; Mataró *et al*., 2001; Frässle *et al*., 2014).

Third, the AIRT states that consciousness is neurophysiologically realized as what Prinz (2012) calls gamma vector waves. According to this conjecture, attending to an object causes the distributed intermediate-level neural populations that encode the various features of that object to become synchronized via enhanced and phased-locked oscillations in the gamma band—a phenomenon referred to as a gamma vector wave. In addition, cells for qualitatively different features, such as shape and color, are proposed to have separate spiking patterns that become coordinated at the coarser time scale of gamma. “By analogy,” writes Prinz (2012, p. 141), “imagine playing two melodies on two different radios while raising and lowering their volume in sync. Each melody would remain intact, but they would now also be heard as parts of the same overarching sound pattern.” This intriguing idea is bolstered by a large body of data, but like the GNWT and the IIT, it has several shortcomings. Most critically, during the conscious observation of recognizable objects, gamma responses are manifested in ways that violate the theory's predictions. For instance, in the mid-level areas that putatively subserve visual awareness, gamma responses do not reliably correlate with subjective reports (Aru *et al*., 2012a). Moreover, as noted above in the discussion of the GNWT, gamma responses increase greatly not only in mid-level areas but also in many high-level areas, some of which contribute to conceptual knowledge and hence should *not* display any neurophysiological signatures of consciousness (Fisch *et al*., 2009; Gaillard *et al*., 2009). Thus, it remains mysterious how the brain regions that underlie our conscious perception of the visual world are operationally distinguished from those that underlie our unconscious understanding of that world.

In sum, because the AIRT adopts the conservative view that concepts never reach awareness, it may have a significant advantage over both the GNWT and the IIT. In addition, it synthesizes in a coherent manner a great deal of empirical and theoretical work in philosophy, psychology, and neuroscience. It is not without limitations, however. Most notably, although the gamma vector wave proposal has several virtues, it—like all other attempts to pinpoint the neurophysiological signatures of consciousness—cannot explain all of the available data.

## Conclusion

To elucidate the neural substrates of consciousness, it is first necessary to determine which mental representations in the flow of information processing do and do not reach awareness. The results of such an analysis can then be used to constrain the psychological phenomena for which unique neural correlates are sought.

According to the liberal view, the contents of experience include not only sensory, motor, and affective states, but also concepts and the thoughts they enter into. This view matches many people's intuitions. For instance, we spend much of our lives producing and comprehending language (both overtly and as inner speech), and it often seems as if these experiences are the equivalent of thinking. In addition, we are accustomed to recognizing objects and events quite rapidly and effortlessly, so it seems natural to suppose that we are directly aware of their meanings. But even though these considerations give some intuitive appeal to the liberal view, I have argued that a variety of other factors strongly favor the opposite conservative view, which maintains that concepts lack intrinsic qualia and always perform their functions beneath the surface of awareness. According to this alternative position, when we process spoken language, the only representations that reach awareness are the pronunciations of words, and they serve as conscious “handles” for the concepts that remain unconscious; likewise, when we recognize objects and events, the only representations that reach awareness are the superficial appearances of stimuli, and, again, they serve as conscious “handles” for the concepts that remain unconscious (Jackendoff, 2012).

If, as I suspect, the conservative view is correct, it will be necessary for future research on the neuroscience of consciousness to distinguish between two levels of representation that are often engaged simultaneously in the brain: First, the kinds of sensory, motor, and affective representations that do reach awareness; and second, the kinds of conceptual representations that do not. I have shown that two of the most prominent and influential theories—namely, Dehaene's GNWT and Tononi’s IIT—fail to draw this distinction because they assume the liberal view. And although a different framework—namely, Prinz's AIRT—does attempt to make the contrast, it unfortunately cannot account for all the available data.

As this field of inquiry continues to advance, it will be essential for investigators to think more deeply about the critical question of whether we are ever aware of concepts. After all, even though I am admittedly biased toward the conservative view, I would be remiss if I did not acknowledge, once again in closing, that the debate between this view and the competing liberal view is by no means over. Indeed, the relevant literature contains far more issues and arguments than I have covered here. My hope is that more neuroscientists will begin to take a greater interest in this literature, and that their experimental and theoretical work will benefit from having done so.
